# Feasibility of engaging “Village Doctors” in the Community-based Integrated Management of Childhood Illness (C-IMCI): experience from rural Bangladesh

**DOI:** 10.7189/jogh.08.020413

**Published:** 2018-12

**Authors:** Sk Masum Billah, DM Emdadul Hoque, Muntasirur Rahman, Aliki Christou, Ngatho Samuel Mugo, Khadija Begum, Tazeen Tahsina, Qazi Sadeq-ur Rahman, Enayet K Chowdhury, Twaha Mansurun Haque, Rasheda Khan, Ashraf Siddik, Jennifer Bryce, Robert E Black, Shams El Arifeen

**Affiliations:** 1 Maternal and Child Health Division, icddr,b, Dhaka, Bangladesh; 2 School of Public Health, University of Queensland, Herston, Australia; 3 Sydney School of Public Health, The University of Sydney, Sydney, Australia; 4 Dalla Lana School of Public Health, University of Toronto, Toronto, Canada; 5 Department of Epidemiology and Preventive Medicine, Monash University, Melbourne, Australia; 6 Department of International Health, Johns Hopkins Bloomberg School of Public Health, Baltimore, Maryland, USA

## Abstract

**Background:**

Informal health care providers particularly “village doctors” are the first point of care for under-five childhood illnesses in rural Bangladesh. We engaged village doctors as part of the Multi-Country Evaluation (MCE) of Integrated Management of Childhood Illness (IMCI) and assessed their management of sick under-five children before and after a modified IMCI training, supplemented with ongoing monitoring and supportive supervision.

**Methods:**

In 2003-2004, 144 village doctors across 131 IMCI intervention villages in Matlab Bangladesh participated in a two-day IMCI training; 135 of which completed pre- and post-training evaluation tests. In 2007, 38 IMCI-trained village doctors completed an end-of-project knowledge retention test. Village doctor prescription practices for sick under-five children were examined through household surveys, and routine monitoring visits. In-depth interviews were done with mothers seeking care from village doctors.

**Results:**

Village doctors’ knowledge on the assessment and management of childhood illnesses improved significantly after training; knowledge of danger signs of pneumonia and severe pneumonia increased from 39% to 78% (*P* < 0.0001) and from 17% to 47% (*P* < 0.0001) respectively. Knowledge on the correct management of severe pneumonia increased from 62% to 84% (*P* < 0.0001), and diarrhoea management improved from 65% to 82% (*P* = 0.0005). Village doctors retained this knowledge over three years except for home management of pneumonia. No significant differences were observed in prescribing practices for diarrhoea and pneumonia management between trained and untrained village doctors. Village doctors were accessible to communities; 76% had cell phones; almost all attended home calls, and did not charge consultation fees. Nearly all (91%) received incentives from pharmaceutical representatives.

**Conclusions:**

Village doctors have the capacity to learn and retain knowledge on the appropriate management of under-five illnesses. Training alone did not improve inappropriate antibiotic prescription practices. Intensive monitoring and efforts to target key actors including pharmaceutical companies, which influence village doctors dispensing practices, and implementation of mechanisms to track and regulate these providers are necessary for future engagement in management of under-five childhood illnesses.

Informal, unqualified health care providers including village doctors, drug sellers, traditional healers and homeopathic doctors are the main source of care for common childhood illnesses in many low-income countries, including the rural and disadvantaged populations in Bangladesh [1-3]. Over 65% of the population of Bangladesh obtain first-line health care services primarily from village doctors [4,5]. A care-seeking study for sick neonates in Bangladesh found that although care was sought for almost 90% of sick neonates, the main providers were homeopaths (38%) and village doctors (37%) [6].

Bangladesh has a pluralistic health system consisting of many different players in the delivery of health services to its population including the government, the private sector, NGOs and bilateral and multilateral donors [7]. The informal private sector in particular has grown rapidly, yet remains unregulated. The formal health workforce is faced with severe shortages of qualified health care providers especially in rural areas [8,9], making it a challenge to meet the demand for health services to a large and expanding population of over 160 million people. Furthermore, the lack of trust in formal health care providers, high out of pocket costs of health care in the public sector, and challenges with accessibility has led to the rapid proliferation of informal health providers to fill the gap between supply and demand across both rural and urban areas [4,9]. These informal health providers now account for 95% of the health workforce in Bangladesh [10]. Village doctors and drug sellers are unlicensed providers of allopathic medicine, most of whom have no or limited standard professional training [9,10]. There are approximately 185 000 village doctors (12.5 per 10 000 population) practicing in the large number of unregistered and unlicensed pharmacies across the country diagnosing patients and selling prescription medicines [7]. Their popularity has persisted over time due to their accessibility and trust they have as members of their communities [10,11].

Village doctors are the predominant health provider to the poor in Bangladesh, and this is unlikely to change in the near future. If they are to continue to provide health care, efforts are needed to improve their practices in the management and treatment of illness. Given the high utilization and numbers of village doctors and the limited resources within the public health sector, there is scope to engage these practitioners in health service delivery. In recognition of their potential, in 1998 village doctors were integrated into the national tuberculosis programme under the country’s national DOTs (Directly Observed Therapy) strategy to improve case detection and treatment of tuberculosis. The success of this initiative led to the involvement of village doctors in the national TB programme becoming part of national policy [12,13]. However, research studies exploring these informal providers’ knowledge and practices in managing various medical conditions, reveals they often incorrectly diagnose illnesses and inappropriately prescribe medications which can be potentially harmful, emphasizing the importance and necessity of training and regular monitoring [14-17].

Very little evidence exists on village doctor’s knowledge and practices regarding the management and treatment of under-five illness, nor the potential for engaging them to accelerate achievements towards reducing child mortality. Pneumonia and other serious infections remain the leading causes of deaths in under-five children in Bangladesh accounting for over a third of under-five child mortality [18,19]. One study in Chakoria, Bangladesh found over 90% of village doctors were involved in the treatment of both diarrhoea and pneumonia, but only 40% of drugs prescribed for pneumonia and 15% for diarrhoea were considered appropriate (according to WHO, UNICEF and Government treatment guidelines) [10]. Greater understanding is required of village doctors’ knowledge and care practices for under-five children and how they can best be engaged to improve their management of sick under-five children.

The baseline findings of the Multi Country Evaluation of the Integrated Management of Childhood Illnesses (MCE-IMCI) showed that 40% of sick children were taken to informal unqualified practitioners including village doctor [20]. As a part of the intervention components, MCE-IMCI included a two-day IMCI training for village doctors on the appropriate management and referral of sick under-five children to minimise harmful practices and ensure referral of severe cases to health facilities.

This study aimed to evaluate the village doctor intervention component of MCE-IMCI to involve village doctors in the management and treatment of under-five children in Matlab, Bangladesh following three years of implementation. We sought to explore the feasibility of engaging village doctors in the management and referral of under-five childhood illnesses, and whether through the provision of a short training on IMCI, knowledge and practices could be improved. As part of this evaluation we also investigated the reasons behind caregiver’s preferences to seek care from village doctors.

## METHODS

### Study setting

The MCE-IMCI was designed to assess the effectiveness of IMCI in improving child survival in five countries including Bangladesh [21]. The study was a pair randomized controlled trial consisting of 10 intervention and 10 comparison areas in Matlab sub-district of Bangladesh. Twenty first-level government health facilities and their catchment areas were first paired based on closeness of principal component analysis scores generated from type of facility, geographical location, baseline mortality levels, and catchment population size. After this catchments from each pair were randomized to IMCI (intervention) and usual services (comparison) areas, described in detail elsewhere [21]. Briefly, the IMCI intervention package consisted of three components: health worker training, health systems improvements, and family and community activities [20]. Implementation of interventions began in 2002. In 2003, the community component of the IMCI intervention introduced a modified two-day IMCI training of village doctors and follow up monitoring for appropriate management and referral of sick under-five children to minimize harmful practices. In 2005, the programme deployed an additional cadre of community health providers known as Village Health Workers (VHW) to establish community level management of non-severe pneumonia and diarrhoea [20]. Final evaluation of the project was done in 2007.

### Description of village doctor intervention

Village doctors providing care for ill under-five children in the IMCI intervention areas were identified by local community-based nutrition workers working under the government’s National Nutrition Programme (NNP) and through a baseline household census done in 2000 where households were asked about preferred sources of care for sick under-five children. In September 2003, 198 village doctors were identified and of these 122 were selected to participate in the IMCI training programme. Selection of village doctors was based on the following: i) They were residing and practicing in the MCE-IMCI intervention areas; ii) practicing allopathic medicine; and iii) were regularly treating under-five illnesses at a high volume. In early 2004, we identified an additional 44 village doctors and based on the criteria set in 2003, 22 of these were included. In total, 144 village doctors participated in the village doctor component of the intervention.

A training module on IMCI was developed for village doctors. The module covered: assessment and management of common childhood diseases including acute respiratory infections, diarrhoea, fever, malnutrition and ear problems; information on age-specific feeding recommendations for healthy and sick children; immunization schedule and when to refer sick children. In total, 144 village doctors were trained using the module over two days in two rounds. The first round of training took place during November-December 2003 and the second round in October 2004. Training involved interactive discussions on identifying harmful practices; classroom sessions and demonstrations of appropriate assessment and management of common childhood diseases; and identification and referral of cases of severe illness in under-five children.

Village doctors were provided with a simplified IMCI patient register to record the sick children they managed, logistics including ARI timers for breathing count, thermometers, referral slips to refer severely ill children to public referral facilities, and a structured format for monthly reporting of cases they saw and referred. MCE-IMCI project staff provided direct monitoring and supervision of the village doctors on a quarterly basis between 2004 and 2007 to encourage adherence with the community IMCI management protocol. Village doctors were also required to attend quarterly follow-up cluster meetings to review their performance, challenges they faced and to provide feedback. They were not given any incentive other than the cost of transportation to attend quarterly meetings.

### Evaluation

Evaluation of the village doctor component of the MCE-IMCI intervention used a mixed method design including both quantitative and qualitative data collection at different time points of the interventions. To assess the feasibility of engaging village doctors in C-IMCI our evaluation framework considered four components- i) effect of project input’s (training, supervision and monitoring) on childhood illness management, knowledge improvement and retention ii) prescription practices of trained village doctors, iii) profile of the village doctors and their business network and iv) reasons for preferring village doctors as the source of care. The different data sources and methods used to assess each of these aspects is summarized in **Table 1****.**

**Table 1:** Evaluation framework describing each component and method used for assessing the feasibility of engaging village doctors in C-IMCI

Evaluation component	Key indicators	Data source and data collection method	Timeline
**Effect of village doctor intervention on knowledge of IMCI case management**	Knowledge of management of pneumonia and diarrhea:	Pre-post structured knowledge test	2003-04 (Before and after training)
	Improvement after training, and	End of project knowledge retention test	2007 (End line)
	Retention over the project period		
**Assessment of village doctor’s prescribing practices post-intervention**	Comparison of treatment and prescribing practice of IMCI trained village doctors with untrained village doctors and medically trained providers on:	Household survey on treatment of sick under-5 child in last 2 weeks	2007 (End line)
	IMCI recommended antibiotics for suspected pneumonia;		
	ORS and Zinc for diarrhoea		
**Profile of village doctors and influencers**	Background and demographic characteristics	Structured survey questionnaire	2007 (End line)
	Training		
	Practice characteristics		
	Business network (relationship with pharmaceutical companies)		
**Examination of reasons caregivers preference of village doctors**	Motivating factors for care-seeking from village doctors	In-depth interviews with mothers who sought care for sick under-5 child from village doctors	2007 (End line)

In May 2007, we administered a structured questionnaire to 131 of the 144 trained village doctors (13 could either not be located or practicing anymore) to obtain their background and socio-demographic characteristics, previous trainings received, practice characteristics, and contact with pharmaceutical companies. We assessed the effect of the two-day IMCI training on village doctors’ knowledge of classification and management of sick under-five children with pneumonia and diarrhoea using a short, self-administered written evaluation test. Of the 144 village doctors that participated in the IMCI training, 135 completed both pre- and post- IMCI training tests in 2003-04. Briefly, the test assessed knowledge on signs of pneumonia and severe pneumonia and the correct management of pneumonia and diarrhoea at home, as well as when it would be necessary to refer a severely ill child and administer a first dose of antibiotics. Out of the total 144 trained in 2003-04, 131 were still practicing during end of project in 2007. The same post-evaluation test was repeated with 38 randomly selected village doctors (25% of IMCI-trained village doctors who were still practicing) to assess knowledge retention.

The prescribing practices of village doctors for the management of childhood illness were assessed in 2007 using an end-of-project MCE-IMCI evaluation census of all households in the programme catchment [20]. All reported sources of care for any under-five illness episode in the two weeks preceding the survey were recorded and used in the analysis. We assessed all providers’ management practices for diarrhoea and pneumonia by requesting caretakers to either to show the prescription or drug packets/bottles or to describe (if prescription or drug not available) the drug prescribed by source of care for the illness. If care was sought from a village doctor, the name and address of their practice location were recorded. Afterwards, source of careseeking was coded as either “IMCI trained” or “untrained village doctors” and this was matched with the list of the village doctors that received the IMCI training. In June 2007, we conducted in-depth interviews with 20 mothers of recently sick children who sought care from a village doctor during the final round of rolling household surveys (January - June 2007). The purpose of the qualitative interviews was to identify factors that influenced caregivers decision to seek care from village doctors over formal medical providers.

### Data analysis

Quantitative data were analyzed using the statistical program STATA Version 13 (Stata Corp, College Station, TX, USA). Descriptive statistics were used to describe the background and practice characteristics of the village doctors, and sources of care for under-five illness and adherence to the MCE-IMCI study monthly reporting and monitoring systems and attendance in quarterly meetings. The McNemar’s test was used to examine changes in village doctors’ knowledge before and after training and knowledge retention. χ^2^ tests, adjusted for clustering, were used to compare prescription practices for pneumonia and diarrhoea management between the different health care providers. Qualitative data from in-depth interviews were transcribed and analyzed using Atlas Ti (ATLAS.ti Scientific Software Development GmbH, Berlin, Germany).

### Ethical considerations

The International Centre for Diarrhoeal Disease Research, Bangladesh (icddr,b) ethics committees reviewed and approved the research study. All study participants provided informed consent. The MCE-IMCI study is registered with a registration number ISRCTN52793850.

## RESULTS

### Demographic and practice characteristics of village doctors

The background characteristics of the 131 IMCI-trained village doctors are summarized in **Table 2**. Half (53%) had between 5-10 years of schooling, and over one-third (36%) had completed 11-12 years of schooling. Over half (57%) practiced from their own drug store, while one third (32%) reported practicing either from home or their drug store, and the majority (89%) practiced during both morning and evening hours. Half were also engaged in work other than their private practice, and the majority (76%) owned a cell phone. Very few (7%) charged consultation fees in addition to the cost of drugs when providing services to ill under-five children. Almost all (94%) village doctors responded to home calls, and less than half (45%) charged fees for these. On average, village doctors had 14 years of experience with managing sick children. At the start of the intervention in 2003-04, 62% (144 out of 232) of all practicing village doctors in the IMCI intervention area were provided with IMCI training. Over the three-year follow-up period, 13 of these IMCI-trained village doctors moved out of the intervention area or stopped practicing, while 78 additional new village doctors started practicing. As a result, by 2007 44% (131 out of 297) of village doctors were trained in IMCI.

**Table 2:** Socio-demographic and practice characteristics of village doctors* that participated in the 2-d IMCI-training, Matlab, Bangladesh as reported in 2007

Characteristic	% (n)
**Age (years)** (N = 131)	
≤30	20.6 (27)
31-40	26.0 (34)
41-50	27.5 (36)
51-60	17.6 (23)
61-70	8.4 (11)
**Education** (N = 131):	
Class 5-10	53.4 (70)
Class 11-12	35.9 (47)
Graduate & above	10.7 (14)
**Monthly income (Bangladesh Taka/BDT)** (N = 131):	
≤3000	10.7 (14)
3000-5000	29.0 (38)
5001-10 000	47.3 (62)
>10 000	13.0 (17)
**Location of chamber** (N = 131):	
Own house	6.9 (9)
Own drug shop	57.3 (75)
Own house and drug shop	32.1 (42)
No longer has a chamber/does not see patients anymore	3.8 (5)
**Timing of practice** (N = 126):	
All day	8.7 (11)
Both morning and evening	88.9 (112)
Only at morning	0.8 (1)
Only at evening	1.6 (2)
**Accessibility:**	
Engaged in work other than health service (N = 126)	50.8 (64)
Owns a mobile phone (N = 131)	76.3 (100)
Attends home calls (N = 126)	93.7 (118)
**Consultation fees** (N = 126):	
Does not take fees when seeing under-five children at practice	92.9 (117)
Takes fee when attending home calls	45.2 (57)
**Years of experience as a Village Doctor** (N = 117)†	
**Years of experience of managing sick children** (mean±SD)	13.9 ± 10.1 y
5 years or less	21.3 (25)
6-10	29.9 (35)
11-15	16.2 (19)
16-20	10.3 (12)
21 years or more	22.2 (26)

SD – standard deviation

*Village doctors participating in IMCI orientation training in 2003-2004 and interviewed in May 2007.

†14 village doctors did not report the duration of service.

**Figure 1:**
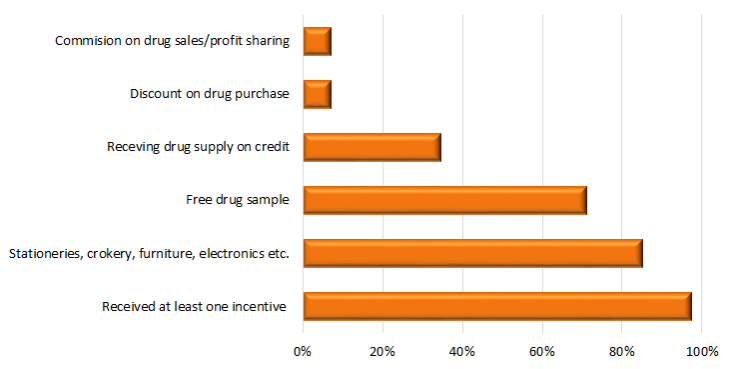
Incentives received by IMCI-trained village doctors from pharmaceutical company representatives as reported in 2007 (N = 115).

Most (86%) IMCI-trained village doctors had received at least one training prior to the IMCI training including a range of private and semi-formal trainings from different sources (**Table 3**). Half had completed the Rural Medical Practitioner training (a programme ranging in duration from a few weeks to a few months, but without a government accredited curriculum [22]), while a quarter had completed the six-month Local Medical Assistant and Family Planning training (a one year training programme more in depth than the RMP that also includes family planning and has no formal accreditation [22]), and/or Village Practitioner or “Palli chikitsok” training, (a government supported training implemented in the late 1980s [9]). A high proportion (80%) reported receiving training or orientation from pharmaceutical companies, most commonly on the use of oral rehydration solution and zinc (49%) and antibiotics (20%). Most village doctors (91%) reported being visited often by pharmaceutical company representatives, and almost all (97%) received at least one incentive or promotional commodity including free drug samples (70%) and gifts including stationary, crockery, and furniture (85%) during these visits (Figure 1).

**Table 3:** Previous training received by village doctors prior to participating in the 2 d IMCI training as reported by village doctors in 2007

Training type and institution	% (n)
Training type and/or certification completed* (N = 113)	
Rural Medical Practitioner (RMP)† training	49.7 (56)
Local Medical Assistant and Family Planning (LMAF)‡ training	24.8 (28)
Village Practitioner training¶	23.9 (27)
Medical Assistant training /Diploma in Medical Faculty (DMF)§	3.5 (4)
Training on specific disease/management (diarrhoea, tuberculosis, malaria) ||	13.3 (15)
Pharmacist	5.3 (6)
Awareness training	4.4 (5)
Training on primary health care	1.8 (2)
Family planning and birth control	1.8 (2)
Other	8.9 (10)
***Any training provided by pharmaceutical companies*** (N = 131)	80.2 (105)
Type of training received from pharmaceutical companies* (N = 105):	
Training on IMCI diseases	5.7 (6)
Training on non-IMCI diseases	6.7 (7)
Training on use of IMCI antibiotics	19.1 (20)
Training on use of non-IMCI antibiotics	19.1 (20)
Use of ORS and/or zinc	48.6 (51)

DMF – Diploma in Medical Faculty, IMCI – Integrated Management of Childhood Illnesses, LMAF – Local Medical Assistant and Family Planning, ORS – Oral Rehydration Therapy, RMP – Rural Medical Practitioner

*Multiple responses possible.

†RMP is a short 6-month programme which trains practitioners on six areas (anatomy, pathology, surgery, ob-gyn, pharmacology and medicine) and provided by different semi-formal institutions with no formal accreditation [22].

‡LMAF is a 1-year training programme more in depth than the RMP that also includes family planning and administered by different private institutions and has no formal accreditation [22].

¶Village Practitioner or “Palli Chickishok” training was an one year training programme for rural practitioners in 1980s supported by the government and was discontinued later [9].

§Medical Assistant/Diploma in Medical Faculty (DMF) training refers to a three-year long training provided by both private and public institutions [15].

||National tuberculosis programme or training organized by district or sub district hospitals under different programme.

### IMCI knowledge improvement and retention

Results from the pre- and post-training knowledge tests in 2003 showed that village doctors’ knowledge on the danger signs of pneumonia and severe pneumonia increased significantly from 39% to 78% (*P* < 0.0001) and from 17% to 47%, (*P* < 0.0001) respectively (**Table 4**). The proportion of village doctors that knew the correct management of severe pneumonia also significantly improved between pre and post-test (62% to 84%; *P* < 0.0001), as did the correct home management of childhood pneumonia (36% to 76%; *P* < 0.0001). Knowledge on the referral of severely ill children which was already high at baseline, still improved significantly after training (**Table 3**). The proportion that knew the signs of severe pneumonia increased slightly from 50% in 2003 to 55% in 2007, as did knowledge on the correct management of severe pneumonia, which rose from 90% to 97%. Correct knowledge of managing persistent diarrhoea was also higher in 2007 compared to 2003 (94% vs 85%). Although not significant increases, these suggest retention of knowledge over time. There was, however, a statistically significant decline from 74% in 2003 to 50% in 2007 (*P* = 0.02) in knowledge on the correct management of pneumonia at home (encouraging continuous uptake of food and fluid, treatment with oral antibiotics, preferably cotrimoxazole, and assessing danger signs).

**Table 4:** Change in knowledge and knowledge retention on the correct management of sick under-five children by village doctors following IMCI orientation training

	Change in knowledge (pre-post) (N = 135 pairs)			Knowledge retention (post-EoP test) (N = 38 pairs)		
**Pre- training (2003)**	**Post-training (2003)**	***P*-value**	**Post-training (2003)**	**EoP (2007)**	***P*-value**
	**% (n)**		**% (n)**	**% (n)**		**% (n)**
% of village doctors who know signs of pneumonia*	39.3 (53)	77.8 (105)	<0.0001	76.3(29)	68.4 (26)	0.405
% of village doctors who know signs of severe pneumonia	17.0 (23)	47.4 (64)	<0.0001	50.0 (19)	55.3 (18)	0.593
% of village doctors who know correct management for severe pneumonia	62.2 (84)	83.7 (113)	<0.0001	89.5 (34)	97.4 (37)	0.083
% of village doctors who know correct management for pneumonia at home†	35.6 (48)	76.3 (103)	<0.0001	73.7 (28)	50.0 (19)	0.020
% of village doctors who know correct management for persistent diarrhoea	65.2 (88)	82.2 (111)	0.0005	89.5 (34)	96.8 (33)	0.706
% of village doctors who know all four danger signs	28.9 (39)	81.5 (110)	<0.0001	81.6 (31)	68.4 (26)	0.095
% of village doctors who know when to refer a severely sick child with first dose of antibiotics	89.6 (121)	99.3 (134)	0.0008	97.4 (37)	94.7 (36)	0.564

EoP – end of Project

*Signs of pneumonia (Fast breathing according to age).

†Management for pneumonia at home (Home treatment with cotrimoxazole/amoxicillin).

Adherence of IMCI-trained village doctors to the programme interventions including participation at cluster meetings and submission of monthly reports were not consistent. Village doctor’s attendance in quarterly meetings varied from 25% to 57%, and submission of monthly reports from 28% to 51%.

### Comparison of prescribing practices for pneumonia and diarrhoea

Prescribing practices for the management of suspected pneumonia and diarrhoea in under-five children by IMCI trained village doctors were compared to other “untrained” village doctors, and with medically qualified providers (**Table 5**). Use of IMCI recommended antibiotics for pneumonia management by IMCI-trained village doctors was higher compared to their untrained peers but the difference was not statistically significant (46% vs 35%; *P* = 0.222). Prescription of higher generation antibiotics for pneumonia management did not vary between the IMCI-trained and untrained village doctors (12% vs 10%; *P* = 0.675). Compared with qualified medical providers, the IMCI-trained village doctor’s performance was significantly lower for pneumonia management (prescription of IMCI recommend antibiotic, 70% vs 46%; *P* = 0.008). IMCI-trained village doctors prescribed non-antibiotic medication (eg, cough syrup for pneumonia and vitamins) significantly more frequently than medically qualified providers for the management of pneumonia (42.5% vs 18.5%, *P* = 0.006) and diarrhoea (16.7% vs 39.0%, *P* = 0.001).

**Table 5:** Comparison of prescription practices for childhood pneumonia and diarrhoea between IMCI-trained village doctors and untrained village doctors and medically qualified providers in 2007

	Type of provider % (n) of sick under-five children for whom care was sought			*P*-values	
Pneumonia (suspected)* Management	**IMCI-trained Village Doctors (A)**	**Untrained Village Doctors (B)**	**Medically Qualified providers**† **(C)**	A vs B	A vs C
IMCI recommend antibiotic‡	45.8 (27)	34.9 (22)	70.4 (38)	0.167	**0.007**
Higher generation antibiotic§	11.9 (7)	9.5 (6)	11.1 (6)	0.678	0.901
Other treatment (Non-antibiotic)	42.4 (25)	55.6 (35)	18.5 (10)	0.145	**0.005**
Total	100 (59)	100 (63)	100 (54)		
**Diarrhoea management:**					
ORS	36.4 (43)	29.4 (45)	45.8 (33)	0.075	0.200
ORS-zinc	2.5 (3)	5.2 (8)	6.9 (5)	0.267	0.143
Antibiotic	22.0 (26)	24.8 (38)	30.6 (22)	0.621	0.190
Other treatment (Non-antibiotic)	39.0 (46)	40.5 (62)	16.7 (12)	0.515	**0.001**
Total	100 (118)	100(153)	100 (72)		

*Suspected Pneumonia: having cough and rapid or difficult breathing not because of blocked nose.

†Medically qualified providers: nurses, paramedics, Sub-Assistant Community Medical Officer (SACMO), Family Welfare Visitors (FWVs); MBBS doctors either prescribed during consultation at public health facility or during private practice).

‡IMCI recommend antibiotic, eg, cotrimoxazole/amoxicillin.

§Higher generation antibiotic – Third generation cephalosporin eg, ceftriaxone/cefixine.

### Factors influencing mothers’ preference to seek care from village doctors

In-depth interviews with mothers of ill under-five children revealed a range of factors that influenced care seeking from village doctors over other available health care service providers. Almost all mothers reported that they were acquainted with the village doctors for a long period of time and the treatment received from the village doctors always worked well. Other reasons behind the preference for village doctors included perceived “good behavior”, considerable flexibility in timing of care provision, longer consultation time and attention, and provision of perceived good and “powerful medicines” during the first visit. As demonstrated by one mother’s comment, *“I prefer going to village doctor who is always available, gives syrup and other medicines that works faster.*”

Health system factors that influenced mothers to choose village doctors over public health facilities included: bad experiences in the past; long waiting times; often high out of pocket expenses including travel cost; inability of the accompanying person to stay at the hospital; and unavailability of drug supply. As one mother stated, “*at health facility we have to wait for long time, they give slip and ask for buying medicines from medicine shop. Why should we go to government hospital crossing a long distance if we have to buy medicine from pharmacy?”* However, almost all the mothers reported that they would go directly to sub-district or district level government hospitals if the condition of the child was serious.

## DISCUSSION

This study has demonstrated that village doctor’s knowledge on the identification and management of under-five childhood illnesses can be improved through the provision of simplified two-day IMCI training program, alongside regular, ongoing supervision and monitoring, and that this knowledge can be retained long term. However, harmful prescribing practices of IMCI-trained village doctors were not reduced compared with non-IMCI trained village doctors over the three-year period, suggesting that adherence with recommended guidelines for the treatment for pneumonia and diarrhoea in under-five children will require broader strategies to address the array of factors that influence their practices. The only knowledge indicator that showed a statistically significant decline after three years was home management of pneumonia with cotrimoxazole or amoxicillin.

Despite improvement of child health care provision at first-level health facilities [21] and the introduction of community-based workers by the programme for delivering curative care for pneumonia and diarrhoea, village doctors remained the dominant and first contact of care for the majority of under-five childhood illness even at the end of the project [20]. They were more accessible to communities in terms of proximity to households and convenience, provided services around the clock and were willing to make house calls, often without charging fees. Similar findings were reported in a study done in southeast Bangladesh [10]. Additional reasons for choosing village doctors as the source of care, reported by mothers in our study, included their unrestricted drug prescription practices and potential to prescribe what was perceived as “strong” antibiotics (eg, second or third line antibiotics) in the first visit, and the flexible payment system available. Similar to other studies, we also found families turned to village doctors because of the poor attitudes of providers, perceived inadequacies of drugs, and higher out of pocket expenses at public outpatient services [23], particularly in cases of serious illness [24]. It is therefore not surprising that village doctors garner such high levels of acceptance and trust in their communities. Although our study was done in 2007, the situation today remains the same in Bangladesh – village doctors continue to be the most available and dominant source of care, in both urban and rural contexts [25], yet few interventions or efforts have been developed to manage or regulate these providers. Limitations in existing public health services, particularly in rural Bangladesh, contribute to the popularity of village doctors because people utilise their services as a complement to the formal health system. Without substantial improvements in services provided by government health facilities, it is unlikely that a shift to greater utilization of the public sector will take place in the near future. Instead, the evidence shows that increasingly people are also turning towards the growing formal private health care sector [19].

The IMCI-trained village doctors in our study were not provided with any incentive to change or improve their practices or adhere to participation in monthly cluster meetings, maintaining patient registers and following guidelines for prescription practices. The competition created by the strong financial and other incentives from pharmaceutical companies attempting to influence the drugs village doctors will prescribe is an important continuing challenge. Village doctors rely on the drugs they sell as their source of income and rarely charge consultation fees, so applying restrictions to what they can their prescribe, as our study did, reduces their profits. This may partly explain why no differences were observed in the prescribing practices between IMCI-trained and untrained village doctors. Further exploration of the motivation behind village doctors prescribing behaviour is needed. Behaviour change itself is a process that takes time and is influenced by an array of factors [26]. Although we provided village doctors with regular monitoring and supportive supervision over the three-year period alongside the initial 2-day IMCI training, this may not have been sufficient, without consideration of external contextual factors. Future research and interventions are required to understand the complexities of these interacting factors.

The relationship between village doctors and pharmaceutical medical representatives is a complex one. Pharmaceutical companies regularly send their representatives to informal providers who exert their influence through incentives. Exploring the interactions between village doctors and pharmaceutical medical representatives (MRs) in Chakoria, Bangladesh, Rahman et al (2014) found that MRs control information that village doctors receive, particularly about new medications, and can influence their practices [27]. It is therefore unlikely that training and engagement alone will alter the influences of these external actors. Future interventions will need to target the actions of these representatives [10]. This will require the involvement of government agencies that oversee drug and policy regulations in the country and pose legal challenges beyond the health sector alone. Engaging pharmaceutical companies to educate and motivate village doctors to reduce the irrational use of antibiotics and increase referral of severely ill cases to health facilities, in addition to promoting drugs, is warranted given that village doctors remain first choice for care for most childhood illnesses in rural settings [28]. Such measures should only be considered as an interim intervention, while a substantial level of effort should be directed to strengthening health services at the community level to divert the largest market share from village doctors to formal health care providers.

The lack of adherence by village doctors with reporting and monitoring activities was a challenge in our study and suggests that if any programme attempts to integrate them as a component of health service delivery, mechanisms to address this issue will be required. A comprehensive review of effective interventions with informal providers in several low- and middle-income countries found that those that were more successful went beyond training alone and altered accountability and incentives, provided feedback, and ongoing monitoring on performance [29]. These are important considerations for the Bangladesh context as well and should be explored.

There are several broader health system issues that need to be addressed in Bangladesh to curb the inappropriate practices of village doctors. Currently, regulations governing health care provision are not implemented in the private and informal sector, and antibiotics and other drugs can be purchased easily without a prescription. This makes it challenging to control prescribing practices of informal providers and potentially endangers the health of children through prescription of harmful drugs and contributes to antibiotic drug resistance as a result of over-prescribing of antibiotics.

Although our study trained village doctors to refer severely ill children to a health facility, by the end of the project we observed an overall decline in the number of referrals made by the IMCI-trained village doctors. It is possible that caregivers refused to comply with referral due to past unsatisfactory experiences or other similar reasons highlighted by previous research [21,30]. Some village doctors in our study reported to the quarterly review teams that they were less inclined to refer because caregivers who complied with referrals often faced unsatisfactory behavior and treatment from service providers at health facilities, which can in turn impact on village doctor’s reputation in the community. Further exploration is needed to understand the motivation underlying village doctors’ decision to refer. And if village doctors are to be engaged and trained to refer severely ill under-five children to public facilities in the future, it will be critical to ensure these facilities are well equipped and resourced with trained staff, and provide a patient-friendly environment so that community members are not deterred from attending.

Despite our best attempts to identify the majority of village doctors with high patient volume in the intervention area, by the end of the study we found a higher proportion of services were sought from village doctors that we had not trained. This may be due to the fact that over the three-year period, the number of practicing village doctors in the intervention area grew by over a third. This reflects the challenges of working with the informal sector, as these providers are inherently difficult to track and identify due to their extremely large and fluctuating numbers and the absence of regulatory mechanism to oversee their practices. The extensive resources required to monitor, train and regulate this vast and ever-growing number of informal providers will be the greatest challenge in working with them. To achieve high coverage of services by trained village doctors, future efforts will need to consider robust and innovative strategies for the continuous identification, training and monitoring of village doctors. Formal and informal professional networks and pharmaceutical companies could be potential sources for identification of village doctors and engaging them in community-based health service delivery. To ensure adherence with rational prescribing practices, substantial efforts will be needed to remove competition for financial benefit fueled by pharmaceutical companies and that will require government effort to regulate the pharmaceutical sector.

### Study strengths and limitations

A major strength of this study is that it is one of the first to conduct long-term follow up village doctors to document the experience of their engagement in formal, community-based management of childhood illness. It provides much-needed evidence for government to initiate action on, and further consideration around how to manage the proliferating informal sector in the country. Our study had some limitations; we did not include direct observation of village doctors’ case management of under-five children and so could not assess the quality of care. The information collected on village doctors’ performance such as training, personal characteristics, and referral of under-five cases was based on self-report. The sample of IMCI-trained village doctors included in the end of project knowledge assessment was small and may have reduced the power to detect statistically significant differences in some of the outcome measures. Despite this limitation, this is also the first study to attempt to report on long-term retention of knowledge among these informal providers in Bangladesh. Although the data from this study was collected over a decade ago, little has changed in how village doctors practice, their levels of training, and so the findings remain relevant for the context in present Bangladesh.

## CONCLUSIONS

Village doctors have enabled greater access to care and medicines particularly to the poor, complementing the gap of the routine health systems especially in rural areas. However, the risks they pose in terms of inappropriate and potentially harmful medical care cannot be ignored, and need to be addressed. Our evaluation of the feasibility of engaging village doctors in C-IMCI demonstrated that their knowledge could be improved and retained by training followed by monitoring and supervision. However, the complex dynamics of profit motive by selling drugs and highly incentivised influence of business network with pharmaceutical companies is likely to have an impact on the limited improvements seen in appropriate management practices especially provision antibiotics. Calls for establishment of effective regulatory mechanisms to monitor and improve their performance have been made repeatedly [11,25,31,32] and are also strongly supported by our study findings.

The development of creative, government-supported strategies that also effectively address the numerous challenges with working with these informal providers and linking in the pharmaceutical sector is needed to gain oversight over the rapidly expanding informal health system and to engage village doctors in a way that harnesses their potential and contributes to child health gains in the country.
